# Conclusions in systematic reviews of mammography for breast cancer screening and associations with review design and author characteristics

**DOI:** 10.1186/s13643-017-0495-6

**Published:** 2017-05-22

**Authors:** Smriti Raichand, Adam G. Dunn, Mei-Sing Ong, Florence T. Bourgeois, Enrico Coiera, Kenneth D. Mandl

**Affiliations:** 1 Centre for Health Informatics, Australian Institute of Health Innovation, Macquarie University, Sydney, NSW 2109 Australia; 2 Centre for Big Data Research in Health, University of New South Wales, Sydney, NSW Australia; 3 Computational Health Informatics Program, Boston Children’s Hospital, Boston, MA USA; 4 Department of Pediatrics, Harvard Medical School, Boston, MA USA; 5 Department of Biomedical Informatics, Harvard Medical School, Boston, MA USA

**Keywords:** Mammography screening, Systematic reviews as topic, Competing interests, Bias, Breast cancer

## Abstract

**Background:**

Debates about the benefits and harms of mammography continue despite the accumulation of evidence. We sought to quantify the disagreement across systematic reviews of mammography and determine whether author or design characteristics were associated with conclusions that were favourable to the use of mammography for routine breast cancer screening.

**Methods:**

We identified systematic reviews of mammography published between January 2000 and November 2015, and extracted information about the selection of evidence, age groups, the use of meta-analysis, and authors’ professions and financial competing interest disclosures. Conclusions about specific age groups were graded as favourable if they stated that there were meaningful benefits, that benefits of mammography outweighed harms, or that harms were inconsequential. The main outcome measures were the proportions of favourable conclusions relative to review design and author characteristics.

**Results:**

From 59 conclusions identified in 50 reviews, 42% (25/59) were graded as favourable by two investigators. Among the conclusions produced by clinicians, 63% (12/19) were graded as favourable compared to 32% (13/40) from other authors. In the 50–69 age group where the largest proportion of systematic reviews were focused, conclusions drawn by authors without financial competing interests (odds ratio 0.06; 95% CI 0.07–0.56) and non-clinicians (odds ratio 0.11; 95% CI 0.01–0.84) were less likely to be graded as favourable. There was no trend in the proportion of favourable conclusions over the period, and we found no significant association between review design characteristics and favourable conclusions.

**Conclusions:**

Differences in the conclusions of systematic reviews of the evidence for mammography have persisted for 15 years. We found no strong evidence that design characteristics were associated with greater support for the benefits of mammography in routine breast cancer screening. Instead, the results suggested that the specific expertise and competing interests of the authors influenced the conclusions of systematic reviews.

**Electronic supplementary material:**

The online version of this article (doi:10.1186/s13643-017-0495-6) contains supplementary material, which is available to authorized users.

## Background

Mammography is the most widely used screening technology for detecting breast cancers in asymptomatic women. Since its introduction, the relative harms and benefits of mammography have been the subject of ongoing debate. Both the age at which to begin breast cancer screening and the frequency of screening have been disputed. Conflicting recommendations persist despite decades of interventional and observational studies that are used as the basis for making recommendations [1–4], and cancer screening guidelines generally fail to quantify benefits and harms in a balanced way [5]. For mammography, the debate was renewed in 2009 when the US Preventive Services Task Force (USPSTF) revised their guidelines to initiate biennial screening at 50 years of age instead of 40 [6]. In 2012, the National Health Services (NHS) in the UK recommended mammography once in 3 years to women aged 47–73 years [7]. In late 2015, the American Cancer Society (ACS) updated their guidelines to initiate annual screening at 45 and reduce the frequency to biennial screening at 55 [8]. In early 2016, the USPSTF again examined the evidence and recommended biennial screening for women between the ages of 50 and 74 [9]. Conflicting recommendations about breast cancer screening make it difficult for clinicians and patients to make informed choices about when to start and how often to repeat mammography for women at average risk.

Inconsistencies in the conclusions produced across systematic reviews may reflect the manner in which the reviews were designed and undertaken, or may be related to the expertise and competing interests held by the reviewers. Analyses in other domains have found that financial conflicts of interest can be associated with differences in the interpretation of the results when drawing conclusions and making recommendations [10–12]. Specific to mammography, an analysis of mostly primary studies showed that authors who worked directly in mammography screening were more likely to downplay or reject over-diagnosis than other authors [13]. Another study examining 12 clinical practice guidelines showed that guidelines authored by radiologists or where lead authors had recent publications on diagnosis and treatment were more likely to recommend routine screening [14].

By measuring review design and author characteristics associated with certain conclusions in systematic reviews of mammography, we may be able to identify factors that are associated with inconsistent conclusions. Our aim was to quantify the degree of disagreement in the conclusions of systematic reviews of mammography for breast cancer screening and then measure associations between the conclusions, design characteristics of the reviews, and the professions and financial competing interests of the authors.

## Methods

### Search strategy

We examined systematic reviews of mammography for breast cancer screening published between January 2000 and November 2015. The PubMed database was searched to identify relevant studies using the following search strategy: “(((((guideline*[Text Word]) OR recommend*[Text Word]) OR review[Publication Type])) AND (((((“mass screening”[MeSH Terms]) OR (“early detection of cancer” [MeSH Terms]) OR screen*[Text Word])))) AND (((mammogr*[Text Word]) OR (breast cancer screening[MeSH terms]) OR (mammography[MeSH Terms])))”. We additionally hand-searched the citations of included reviews after screening to identify any other relevant literature.

### Selection of reviews

Review articles were included in the analysis if they met all four of the following inclusion criteria: (a) the reviewers specified a search criteria and the databases in which the search was conducted; (b) the review was focused on mammography for breast cancer screening; (c) at least two primary studies addressing the harms or benefits of mammography were cited; and (d) the reviewers made conclusions about the harms or benefits of mammography for breast cancer screening in relation to the evidence. Outcomes related to benefits included breast cancer survival (mortality reduction), and cost-effectiveness of screening for quality-of-life. Outcomes related to harms included over-diagnosis, false positives, unnecessary treatments, radiation cancers, anxiety or worry, and pain or discomfort. Reviews that examined only diagnosis endpoints without considering survival or harms were excluded, as were reviews that only considered high-risk populations or populations of women who had previously been diagnosed with breast cancer. Articles were also excluded if they were guidelines, no longer archived or accessible online, not peer reviewed, or were in a language other than English.

### Screening and data extraction

Two investigators independently screened article titles and abstracts against the inclusion criteria, and then examined the full text of articles against the inclusion and exclusion criteria. Discrepancies were resolved by discussion at both stages.

The review design characteristics extracted included patient age ranges covered in the evidence, the types of primary studies analysed, the set of outcome measures examined, the presence or absence of a meta-analysis, and the year the systematic review was published. Patient age ranges were assigned to one of four categories: 49 or under, 50 to 69 years, 70 years or older, and one other group for reviews that considered all ages or did not specify an age range. These age groups were selected to correspond with the common age ranges used in the most recent guidelines. The age group for women aged between 50 and 69 in particular is where there has been substantial disagreement about how often women should undergo mammography, and this was a focus of our study. Where age ranges differed from the four groups, we identified the group with the largest overlap (see Additional file 1). The types of studies included in the review were classified into controlled trials, observational studies, both forms of primary studies, or cost-effectiveness analyses.

We recorded the professional role or specialty of all individual authors and categorised them as clinicians or non-clinicians. Professional role was determined by the affiliation listed on the article, employment history, qualifications, and listed research interests. These elements were identified and interpreted from the systematic review and biographies on institutional webpages where available. Qualifications for clinicians included MD or equivalent degrees, and non-clinical qualifications included PhD and MPH. Where corresponding authors had both clinical and non-clinical qualifications, we assigned them to the clinician group if we identified a clinical affiliation on institutional webpages or recent publications, and to the non-clinician group if we could find no such evidence. Where authors were all clinicians or all non-clinicians, we labelled the review as such, and where the authors had a mix of the two types of professional roles, we labelled the review according to the professional role of the corresponding author, under the assumption that the corresponding author takes primary responsibility for the conclusions drawn in the review (Additional file 2).

A financial competing interest disclosure may have described research funding, ownership, or fees from a developer of mammography systems or software. Financial competing interests were determined from disclosure statements in the article. If a competing interest was identified and was financial in nature, we assumed it to be relevant and labelled all conclusions in the systematic review as associated with a financial competing interest. We also noted the presence or absence of a disclosure statement in the systematic reviews and labelled systematic reviews without a disclosure separately.

Two investigators read each included systematic review in its entirety to evaluate the mammography recommendations contained in the conclusions. Each conclusion was judged as favourable or non-favourable by assigning it to one of eight types. Four types were considered to be favourable (evidence of benefits, benefits outweigh harms, the practice is cost-effective, no evidence of harms), and four were labelled as non-favourable (evidence of harms, harms outweigh benefits, the practice is not cost-effective, no evidence of benefits). A third investigator read any reviews for which there was a disagreement to produce a final grading. For each conclusion, we also extracted supporting conclusion statements from the systematic reviews, as well as any statements that made recommendations about who should undergo screening by mammography and how often it should be done.

Some of systematic reviews examined evidence for different age ranges or frequency of screening separately, producing conclusions for each. These conclusions were considered separately. This means that systematic reviews may be represented in the analysis with more than one conclusion and those conclusions may differ.

### Analysis

A linear regression was used to check whether favourable conclusions became more or less common over the period of study, testing whether changes in the primary evidence influenced the likelihood of a favourable conclusion in systematic reviews. Where appropriate, we performed chi-square tests to test the association between favourable conclusions and each of the review design (evidence selection, age groups, outcomes, or the use of meta-analysis) and author (professional roles and financial competing interests) characteristics extracted from the systematic reviews.

## Results

**Fig. 1:**
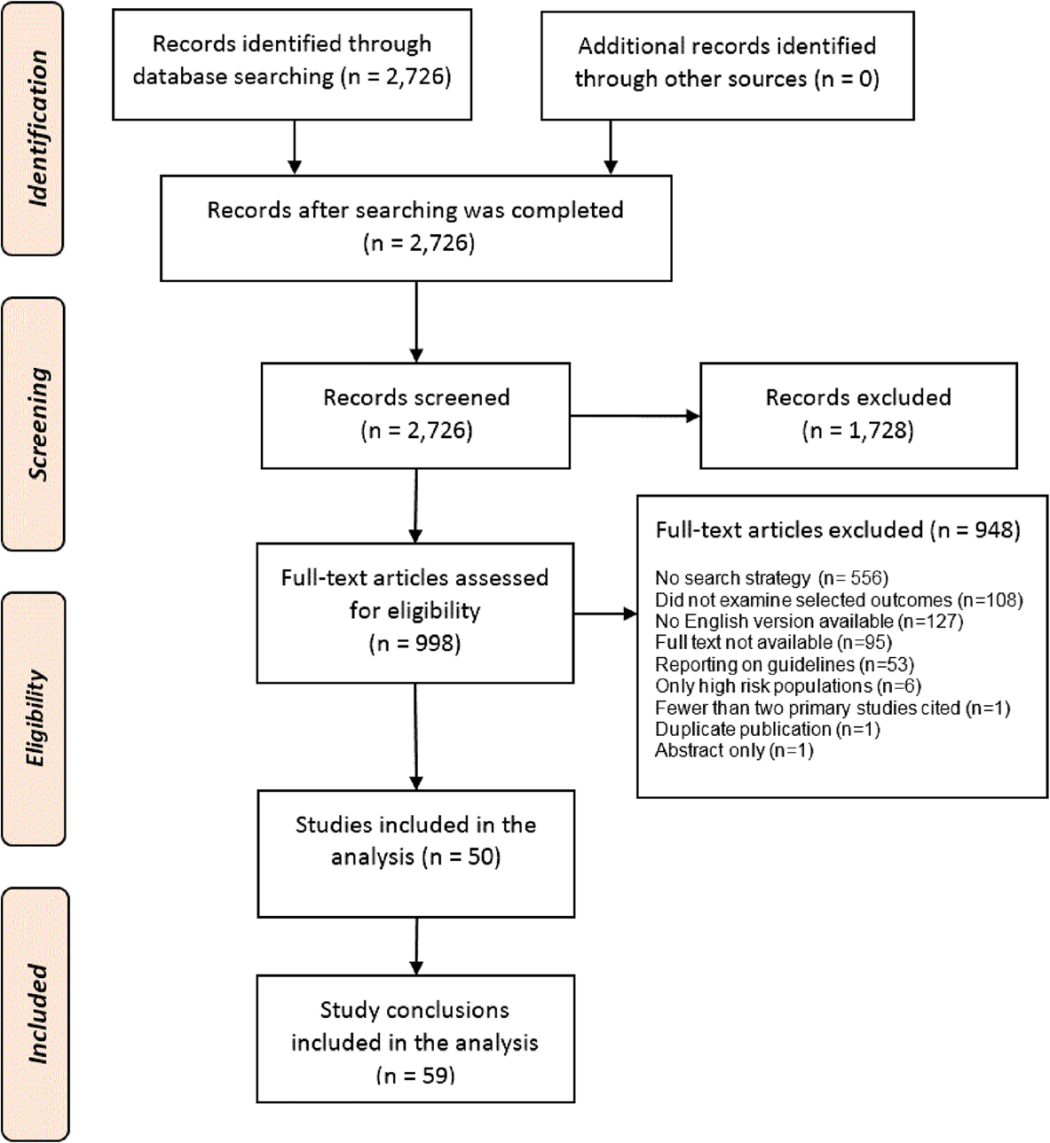
From a search identifying 2726 articles, 59 conclusions from 50 systematic reviews were included in the analysis

The search returned 2726 publications, from which 50 systematic reviews met the inclusion criteria (Fig. 1). Five systematic reviews included separate conclusions for two or more age groups, yielding a set of 59 conclusions and 42 corresponding authors.

### Summary systematic review characteristics

Among the 59 conclusions, 42% (25/59) were graded as favourable. The statements that best characterised the grading are provided as Additional files 3 and 4).

The greatest number of conclusions were for women aged between 50 and 69 (22 conclusions), and for which there was no restriction on age range in the evidence cited or where no age range was specified (also 22 conclusions). Of the remainder, 17% (10/59) considered women under 49, and 8% (5/59) considered women aged 70 and older (see Additional file 5).

Evidence selection also varied across the conclusions. Conclusions based entirely on randomised controlled trials comprised 20% (12/59). Another 41% (24/59) considered both randomised controlled trials and observational studies, 34% (20/59) considered only observational studies, and 5% (3/59) were based on cost-effectiveness studies. Mortality outcomes were used in 64% (38/59) of the conclusions. Meta-analyses supported 27% (16/59) of the conclusions.

Most conclusions were associated with author groups that had a public health, epidemiology, or biostatistics (68%; 40/59) background. Among the 32% (19/59) of corresponding authors from clinical specialties, 6 were from oncology, 4 from radiology, and 10 from other medical specialties (see Additional file 6). Disclosures of relevant financial competing interests were present in 14% (8/59) of conclusions.

**Fig. 2:**
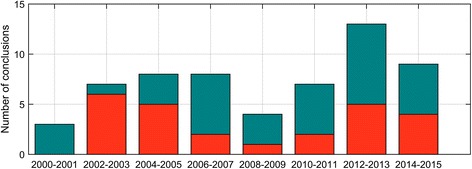
The number of systematic review conclusions by publication years during the period, including favourable conclusions (*orange*) and non-favourable conclusions (*cyan*)

The number of systematic review conclusions produced over the period peaked between 2004 and 2007, and increased again from 2010 (Fig. 2). Fitting a linear regression to the proportion of favourable conclusions per year revealed no clear increase or decrease in the proportion of favourable conclusions over time (*r* ^2^ = 9.73 × 10^−3^; *p* = 0.716).

### Associations between systematic review characteristics and conclusions

Of the conclusions written by corresponding authors from clinical specialties, 63% (12/19) were graded as favourable (Table 1). A greater proportion of the conclusions made in systematic reviews written by clinicians were favourable compared to conclusions by corresponding authors from a public health and epidemiology profession, for which 32% (13/40) of conclusions were graded as favourable. Among the conclusions associated with a disclosed financial competing interest, 75% (6/8) were favourable. In comparison, 47% (9/19) were graded as favourable when there was no disclosure, and 31% (10/32) were graded as favourable when there was an explicit declaration of no financial competing interests. The proportions of favourable conclusions relative to combinations of clinical profession and financial competing interest disclosures revealed a consistent pattern in which corresponding authors who were clinicians or had financial competing interests more often produced favourable conclusions (Fig. 2).

**Table 1:** Characteristics of the review and author characteristics relative to the conclusions

Characteristics	Number of conclusions	Favourable conclusions (% of type)	Non-favourable conclusions (% of type)
Corresponding author			
Non-clinical	40	13 (32%)	27 (68%)
Clinical	19	12 (63%)	7 (37%)
Competing interests			
Declared none	32	10 (31%)	22 (69%)
Declared	8	6 (75%)	2 (25%)
No statement	19	9 (47%)	10 (53%)
Type of evidence			
Both	24	7 (29%)	17 (71%)
RCT only	12	5 (42%)	7 (58%)
Non-RCT only	20	11 (55%)	9 (45%)
Cost effectiveness	3	2 (67%)	1 (33%)
Age groups			
Not specified/all ages	22	7 (32%)	15 (68%)
Up to 49	10	4 (40%)	6 (60%)
50–69	22	10 (45%)	12 (55%)
70 and over	5	4 (80%)	1 (20%)
Outcome measures^a^			
Mortality	38	19 (50%)	19 (50%)
Over-diagnosis	25	8 (32%)	17 (68%)
Specific harms	28	9 (32%)	19 (68%)
Cost effectiveness	8	5 (62%)	3 (38%)
Meta-analysis			
No	43	21 (49%)	22 (51%)
Yes	16	4 (25%)	12 (75%)
Total	59	25 (42%)	34 (58%)

^a^Totals for outcome measures sum to more than the total because systematic review conclusions may have considered more than one outcome

**Fig. 3:**
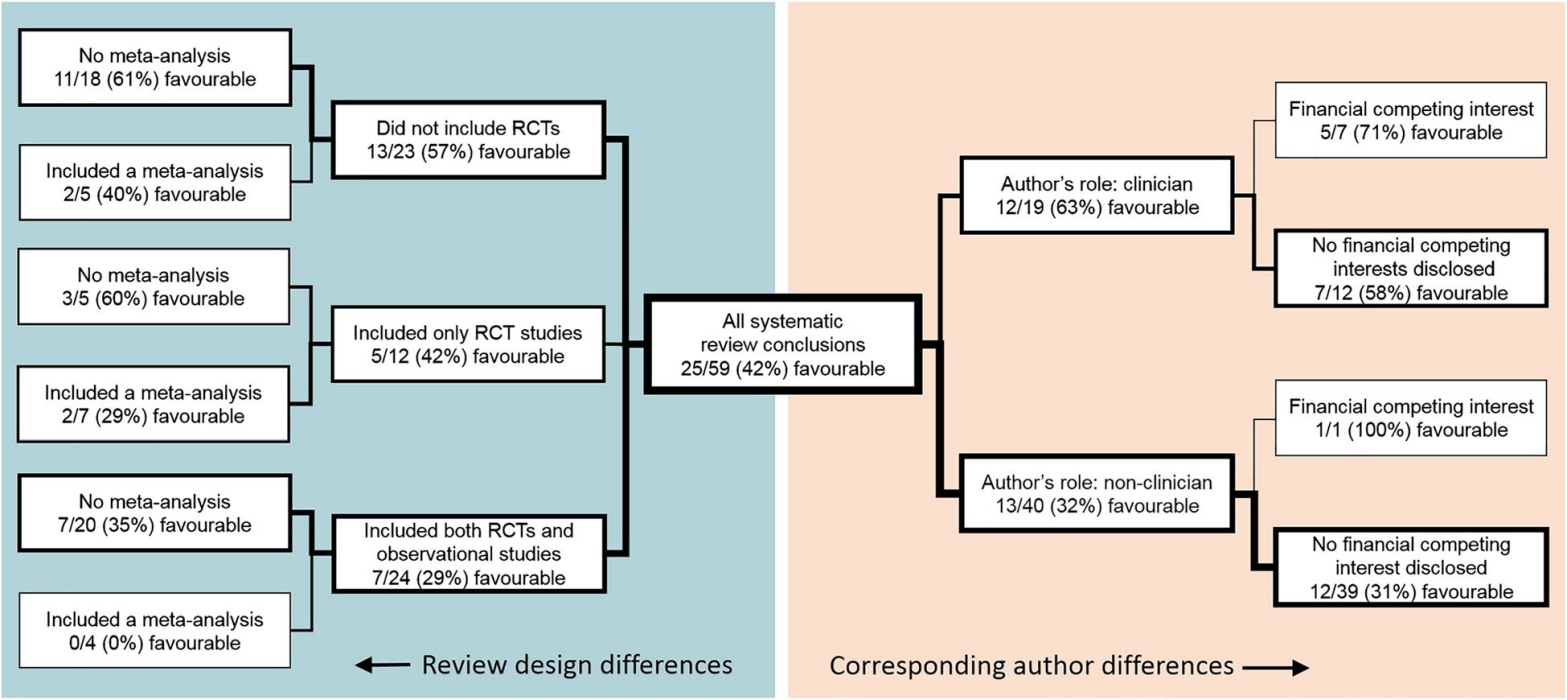
The proportions of systematic review conclusions that were favourable relative to review design and corresponding author differences illustrating some consistent patterns. Line widths correspond to the number of conclusions

Among the conclusions drawn from reviews of randomised controlled trials, 42% (5/12) were graded as favourable; compared to 29% (7/24) of conclusions based on studies of both controlled trials and observational studies; 55% (11/20) of conclusions based only on observational studies; and 67% (2/3) of conclusions based on cost-effectiveness studies (Table 1). For conclusions about women under 50, 40% (4/10) were favourable, 45% (10/22) were graded as favourable for women aged 50–69, 80% (4/5) for women aged 70 and over, and for women of all ages (or where age was not specified), 32% (7/22) were graded as favourable. Among the conclusions drawn in systematic reviews that used a meta-analysis, 25% (4/16) were graded as favourable, compared to 49% (21/43) of those with no meta-analysis (Fig. 3).

For the subset of conclusions relating to women aged 50–69 (corresponding to the age group for which mammography is recommended by several current guidelines), the 20 conclusions were produced by different authors and we were able to more reliably test the association between the observed characteristics and favourable conclusions (Table 2). In this subset, 27% (3/9) of conclusions from non-clinicians were favourable compared to 78% (7/9) from clinicians (OR 0.11; 95% CI 0.01–0.84; *p* = 0.025; *χ* ^2^ = 5.05). When corresponding authors declared no financial competing interests, 20% (2/10) of the conclusions were graded as favourable compared to 80% (8/10) when authors declared a financial competing interest or did not include a disclosure statement (OR 0.06; 95% CI 0.07–0.56; *p* = 0.007; *χ* ^2^ = 7.20). Analyses in other age groups did not indicate a significant difference (see Additional files 5, 7, and 8). We found no clear evidence that the types of primary studies included in the analysis, choice of outcomes, or the use of meta-analysis were associated with favourable conclusions.

**Table 2:** Associations between systematic review characteristics and conclusions for women aged 50–69

Characteristics	Number of conclusions	Proportion of favourable conclusions (%)	*p* value (chi-square test)
Corresponding author			
Non-clinical	11	3 (27%)	
Clinical	9	7 (78%)	
			*p* = 0.025; *χ* ^2^ = 5.05
Competing interests			
Declared none	10	2 (20%)	
No statement	6	4 (67%)	
Declared	4	4 (100%)	
			*p* = 0.016; *χ* ^2^ = 8.27
Type of evidence			
RCT only	5	1 (20%)	
RCT and non-RCT	8	4 (50%)	
Non-RCT only	6	4 (67%)	
Cost-effectiveness	1	1 (100%)	
			*p* = 0.325; *χ* ^2^ = 3.47
Outcome measures			
Did not include harms	9	6 (67%)	
Included harms or over-diagnosis	11	4 (36%)	
			*p* = 0.178; *χ* ^2^ = 1.81
Meta-analysis			
Yes	7	2 (29%)	
No	13	8 (62%)	
			*p* = 0.160; *χ* ^2^ = 1.98

Equivalent conclusions and characteristics for reviews separately considering age groups 50–59 and 60–69 were combined

*RCT* randomised controlled trial

## Discussion

As evidence accumulates, systematic reviews addressing the same clinical question should converge toward a common conclusion. In the last 15 years, 50 systematic reviews on the use of routine mammography for breast cancer screening in asymptomatic women have been published but a consistent conclusion has not emerged. While we did not find strong evidence to suggest that these differences were associated with the types of studies or outcomes included, or the use of meta-analysis, we did find that authors from certain professions were more likely to produce favourable conclusions for women aged between 50 and 69. There were too few conclusions published in systematic reviews for women under 50 and women 70 or older to reliably identify factors associated with favourable conclusions. The results suggest that the expertise and experience of the authors of systematic reviews may have influenced the conclusions in ways that could not be easily identified as differences in the designs of the reviews.

Author professions or specialties have also been associated with conclusions in primary studies and clinical practice guidelines for mammography. For clinical practice guidelines, both author specialty and competing interests were associated with a recommendation of routine screening [14]. In an analysis of mostly primary studies, authors who worked in screening less often included over-diagnosis as an outcome or minimised it as a harm [13]. We found similar results for systematic reviews on the same topic. Together, the results from these studies suggest that one of the reasons why we continue to see ongoing debate about the harms and benefits of mammography may be because the expertise and experiences of the researchers who report the evidence have influenced both the manner in which it is reported and the conclusions and recommendations that have been made.

Previous studies examining differences in systematic reviews on other topics have demonstrated associations between the characteristics of authors and the conclusions they have drawn. These studies have often examined financial and non-financial competing interests [10, 11, 15]. In each case, the analyses demonstrated that authors of systematic reviews may introduce biases in the design and reporting of systematic review to produce conclusions that are aligned with their interests. The introduction of biases into the design and reporting of systematic reviews may be subtle [16, 17], so it may be difficult to quantify where biases are introduced in the systematic review process.

### Limitations

There were several limitations to the analysis we performed. Non-systematic reviews were not considered in the analysis, and it is likely that reviewers with affiliations other than public health and epidemiology would be more commonly represented in non-systematic reviews. Similarly, we did not consider systematic reviews that were focused on the performance of mammography for diagnosis, and practitioners may have been more commonly represented in these systematic reviews. We did not use any formal tools—such as AMSTAR or the PRISMA checklist [18, 19]—to assess the quality of the reviews or the reporting of the reviews on mammography screening, which may have also been associated with differences in the conclusions. However, prior research has found that there were differences in quality among clinical practice guidelines for mammography [20]. We did not investigate financial competing interests beyond what was disclosed by the authors—for some journals, the authors may not have been required to disclose their financial competing interests, which means that we may have under-reported the true rate of financial competing interests. Finally, because some authors were present in more than one review and some reviews included more than one conclusion, certain characteristics may have been over-represented in the sample, so we were limited in what we could conclude from the statistical analyses.

## Conclusions

Analysing 15 years of systematic reviews examining the benefits and harms of mammography for routine breast cancer screening in asymptomatic women, we found that there is still no consensus across systematic reviews about when and how often mammography should be used. Despite the volume of evidence available for women aged 50 to 69, disagreements between guidelines about frequency are common. For this age group, the results suggest that favourable conclusions were more common in systematic reviews written by authors who were clinicians. The results were generally consistent with studies examining primary studies and clinical practice guidelines about mammography for routine breast cancer screening. The results provide further evidence to suggest that conclusions in systematic reviews may be influenced by the expertise and experiences of the authors who write them rather than when they were published, the evidence included, or the designs of the reviews.

## Additional files

Additional file 1: Age group ranges. Age group ranges identified in the systematic reviews and their classification in the analysis. Shaded regions represent the age groups used in the analysis (orange rectangles) and the set of all age groups identified in the systematic reviews are labelled by their reference number and age range (grey rectangles). (PDF 374 kb)

Additional file 2: Table S5. Authors’ professional roles; (C: clinican; N: non-clinician; G: group; triangles indicate the corresponding author). (PDF 602 kb)

Additional file 3: Table S6. Systematic review conclusion and recommendation statements. (PDF 433 kb)

Additional file 4: Included systematic reviews. References for all included systematic reviews. (PDF 229 kb)

Additional file 5: Table S3. Associations between systematic review characteristics and conclusions in 5 conclusions of studies that included women aged 70 years and older. (PDF 193 kb)

Additional file 6: Table S4. Included systematic review conclusions in detail. (PDF 774 kb)

Additional file 7: Table S1. Associations between systematic review characteristics and conclusions in 22 conclusions of studies that did not specify age group or specified all ages. (PDF 193 kb)

Additional file 8: Table S2. Associations between systematic review characteristics and conclusions in 10 conclusions of studies that included women aged up to 49 years. (PDF 193 kb)

## Abbreviations

AMSTAR: Assessment of Multiple Systematic Reviews
MD: Medical doctor
NHS: National Health Service
PRISMA: Preferred Reporting Items for Systematic Reviews and Meta-Analyses
USPSTF: US Preventive Services Task Force
