# Supplementary material for: Conclusions in systematic reviews of mammography for breast cancer screening and associations with review design and author characteristics
Source: Syst Rev. 2017 May 22;6:105. doi: 10.1186/s13643-017-0495-6 (PMC5441061; doi:10.1186/s13643-017-0495-6)
Supplement: Supplementary file 1 — Age group ranges. Age group ranges identified in the systematic reviews and their classification in the analysis. Shaded regions represent the age groups used in the analysis (orange rectangles) and the set of all age groups identified in the systematic reviews are labelled by their reference number and age range (grey rectangles). (PDF 374 kb) [file 13643_2017_495_MOESM1_ESM.pdf]

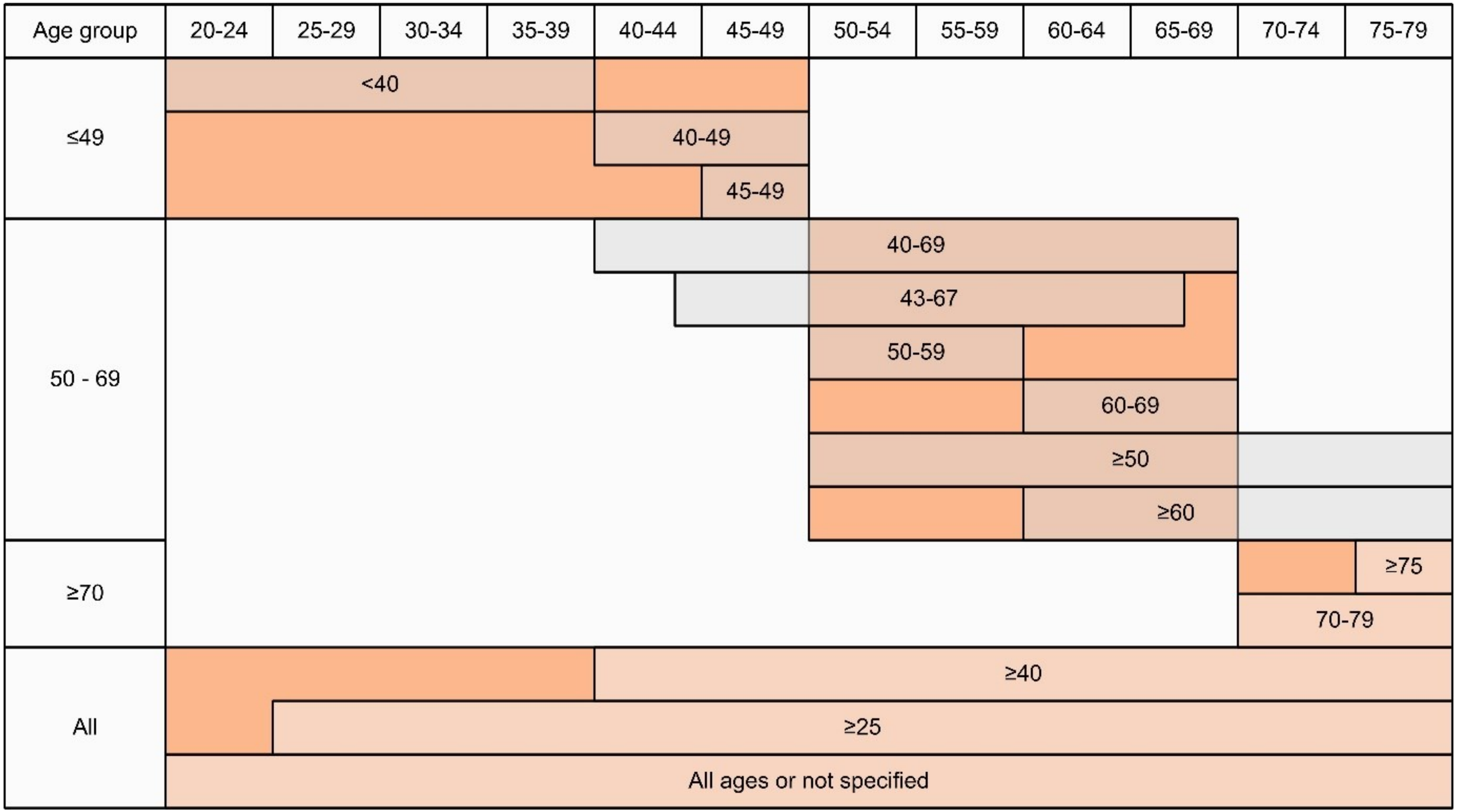

Additional File 1. Age group ranges identified in the systematic reviews and their classification in the analysis. Shaded regions represent the age groups used in the analysis (orange rectangles) and the set of all age groups identified in the systematic reviews are labelled by their reference number and age range (grey rectangles).
