# Supplementary material for: Conclusions in systematic reviews of mammography for breast cancer screening and associations with review design and author characteristics
Source: Syst Rev. 2017 May 22;6:105. doi: 10.1186/s13643-017-0495-6 (PMC5441061; doi:10.1186/s13643-017-0495-6)
Supplement: Supplementary file 2 — Authors’ professional roles; (C: clinican; N: non-clinician; G: group; triangles indicate the corresponding author). (PDF 602 kb) [file 13643_2017_495_MOESM2_ESM.pdf]

Additonal Table 5. Author professional roles (C: clinican; N: non-clinician; G: group; triangles indicate the corresponding author)

Conclusion by systematic review and age group	First author	Second author	Third author	Fourth author	Fifth author	Sixth author	Seventh author	Eight author	Ninth author	Tenth author	Eleventh author
Armstrong, K.; 2007[1] (40-49y)	C▲	C	N	N	C						
Autier, P.; 2011[2]	N▲	N	N	N	N	N	N				
Baker, S.; 2005 [3]	N▲	C	N								
Biesheuvel,C.; 2007[6] (40-49y)	N▲	N	N	N	N						
Biesheuvel,C.; 2007[6] (50-59y)	N▲	N	N	N	N						
Biesheuvel,C.; 2007[6] (60-69y)	N▲	N	N	N	N						
Bond, M.; 2013[7]	N▲	N	N	N	N	N	N				
Bond, M.; 2013[8]	N▲	N	N	N	N	N	N				
Brett, J.; 2005[9]	N	N	N	N	N▲						
Brewer, N. T.; 2007[10]	N▲	N	N								
Elmore, J. G.; 2005[12] (40-49y)	C▲	C	C	N							
Erpeldinger, S.; 2013[13]	N▲	N	C	C	C	N	C				
Gotzsche, P. C.;2000[16]	N▲	N									
Gotzsche, P. C.; 2013[17]	N▲	N									
Gotzsche, P. C.; 2006[18]	N▲	N									
Gotzsche, P. C.; 2009[19]	N▲	N									
Gotzsche, P. C.; 2011[20]	N▲	N									
Gotzsche, P. C.; 2011[21]	N▲										
Hafslund, B.; 2009[23]	C▲	N									
Hamashima, C.; 2015[24] (50+y)	C▲	C	C	N	N	N	C				
Hofvind, S.; 2012[26]	C▲	N	N	N	N	N	N	C	C	G	
Jorgensen, K. J.; 2009[28]	N▲	N									
Jorgensen, K. J.;2013[29]	N▲										
Leung, G. M.; 2002[31]	N▲	N	N	N							
Metsala, E.; 2012[33]	N▲	C	N								
Olsen, O.; 2001[36]	N▲	N									
Pace, L. E.; 2014[37] (40-49y)	C	N▲									
Pace, L. E.; 2014[37] (50-59y)	C	N▲									
Pace, L. E.; 2014[37] (60-69y)	C	N▲									
Paesmans, M.; 2010[38]	N▲	N	N	N							
Puliti, D.; 2012[39]	N	N	N	N	N	N	C▲	G			
Ringash, J.;2001[41]	C▲	G									
Walter, L. C. ; 2014[47] (Biennial)	C▲	C									
Yoo, K. B.; 2013[49]	N	N	N	N	N	N	N	N	N▲		
Armstrong, K.; 2007[1] (50y+)	C▲	C	N	N	C						
Baker, S.; 2005[4]	N▲	C	N								
Barratt, A. L.; 2002[5]	N▲	C	N	N	N						
Broeders, M.; 2012[11]	N▲	N	N	N	N	N	N	N	N	N	G
Elmore, J. G.; 2005[12] (60-69y)	C▲	C	C	N							
Elmore, J. G.; 2005[12] (70+)	C▲	C	C	N							
Gabe, R.; 2005[14]	N▲	N									
Galit, W.; 2007[15]	N	N	N▲								
Green, B. B.; 2003[22]	N▲	N									
Hamashima, C.; 2015[24] (40-49y)	C▲	C	C	N	N	N	C				
Harris, R.; 2011[25]	N▲	N	N								
Jones, B. A.; 2003[27]	N▲	N	N								
Lee, S. J.; 2013[30]	C▲	N	N	C	N	N					
Mandelblatt, J.; 2003[32]	C▲	N	N	N	C	N	C	N			
Moss, S. M.; 2012[34]	N▲	N	N	N	N	N	N	G			
Njor, S.; 2012[35]	N▲	N	N	N	N	N	N	G			
Ravert, P. K.; 2010[40]	C▲	C									
Royak-Schaler, R.; 2002[42]	N▲	N									
Scheel, J. R.; 2015[43]	C▲	C	N	C	C						
Schopper, D.; 2009[44]	N▲	C									
Suzuki, A.; 2014[45]	C	C	C▲								
Tange, U. B.; 2002[46]	C▲	N	C	C	C	C	N				
Walter, L. C. ; 2014[47] (Annual)	C▲	C									
Yarbrough, S. S.;2004[48]	C▲										
Zelle, S. G.; 2013[50]	N▲	N									
