# Supplementary material for: Conclusions in systematic reviews of mammography for breast cancer screening and associations with review design and author characteristics
Source: Syst Rev. 2017 May 22;6:105. doi: 10.1186/s13643-017-0495-6 (PMC5441061; doi:10.1186/s13643-017-0495-6)
Supplement: Supplementary file 3 — Systematic review conclusion and recommendation statements. (PDF 433 kb) [file 13643_2017_495_MOESM3_ESM.pdf]

Additional Table 6. Systematic review conclusion and recommendation statements.

Article and age group	Outcomes	Direction of conclusion	Conclusion statement	Recommendation Statement
Armstrong, K.; 2007 (40-49y)[1]	Mortality; Overdiagnosis; False +ve; Radiation Cancer; Pain/Discomfort	Not favourable	Current evidence indicates that women 40 to 49 years of age who undergo routine mammography screening will decrease their risk for death due to breast cancer but will increase their risks for undergoing unnecessary procedures, breast cancer–related anxiety, discomfort at the time of screening, and exposure to low-dose radiation.	Given this difference, a woman 40 to 49 years of age who had a lower-than-average risk for breast cancer and higher-than-average concerns about false-positive results might reasonably delay screening.
Armstrong, K.; 2007 (50+y)[1]	Mortality; Overdiagnosis; False +ve; Radiation Cancer; Pain/Discomfort	Favourable	The proportion of women 50 years of age or older whose risks for mammography outweigh the benefits is widely accepted to be clinically insignificant.	None
Autier, P.; 2011[2]	Mortality	Not favourable	In contrast, this study found that in general, incidence rates of advanced breast cancer did not change much despite 7–15 years of good participation in mammographic screening....." "....These elements indicate that the lower than expected decrease in advanced cancer incidence seems not attributable to age at screening, to screening frequency or to the way screening is implemented...."	None
Baker, S.; 2005 [3]	Mortality; Overdiagnosis; False +ve; Radiation ; Cancer; Cost effectiveness/ QoL	Not favourable	There is limited evidence for benefit in mortality reduction among 40–49 year old women. The relative risk reduction is estimated at 15% (compared with 22% in women aged over 50), but could be less, depending on the extent to which it is due to screening women after they reached the age of 50. The most recent report suggests a borderline statistically significant 10 or 11% reduction in predicted deaths at 10 years in women invited for screening—less than that observed in many other studies that included women below the age of 50, and less than in either of the meta-analyses reported in this article.	None
Baker, S.; 2005[4]	Mortality	Favourable	Evidence from less robust studies supports a screening interval of one year or 18 months—although in the randomised controlled trials, shorter screening intervals did not produce greater benefits among women aged 45 to 49 years.	They decided, after much discussion, to recommend a two-yearly screening interval for women aged 45 to 49 at this time—taking into account the available evidence and the ability of the health sector to cope with the increased workload.
Barratt, A. L.; 2002[5]	Cost effectiveness/ QoL	Favourable	The benefit of screening women 70–79 years is about 40%–72% of that achieved in women 50–69 years, and declines further with increasing age and when quality-of-life adjustment is made. Extending screening to women 70–79 years is relatively cost-effective (similar to that of extending screening to women 40–49 years of age).	Women may want to decide for themselves whether to continue screening. The choices women make are likely to vary, depending on how each woman values the possible benefits and risks.
Biesheuvel,C.; 2007[6]	Overdiagnosis	Not favourable	On the basis of our review, there is inadequate high quality evidence about overdetection of invasive breast cancer by screening mammography. We conclude that the least biased estimates of overdetection reported so far are those of Zackrisson and co-workers and Moss, based on the Canadian trials. These estimates range from –4% to 7·1% for women aged 40–49 years	None
Biesheuvel,C.; 2007[6]	Overdiagnosis	Not favourable	On the basis of our review, there is inadequate high quality evidence about overdetection of invasive breast cancer by screening mammography. We conclude that the least biased estimates of overdetection reported so far are those of Zackrisson and co-workers and Moss, based on the Canadian trials. These estimates range from 1·7% to 54% for women aged 50–59 years,	None
Biesheuvel,C.; 2007[6]	Overdiagnosis	Not favourable	On the basis of our review, there is inadequate high quality evidence about overdetection of invasive breast cancer by screening mammography. We conclude that the least biased estimates of overdetection reported so far are those of Zackrisson and co-workers and Moss, based on the Canadian trials. These estimates range from 7% to 21% for women aged 60–69 years	None
Bond, M.; 2013[7]	Anxiety	Not favourable	Therefore, it may be reasonable to speculate that, for those in the general population, having a false-positive screening mammogram can cause breast cancer-specific psychological distress that may endure for up to 3 years. However, it is less likely that there will be general anxiety detectable at clinically recognisable levels.	Policy makers, particularly in the UK should consider the impact of false-positive mammograms when planning breast screening services.

Article and age group	Outcomes	Direction of conclusion	Conclusion statement	Recommendation Statement
Bond, M.; 2013[8]	Anxiety	Not favourable	The strongest evidence suggests that the distress caused by a false-positive mammogram may be sufficient to deter an additional 3% of women from attending their next breast cancer screening appointment.	It is important to provide women who are recalled with clear, carefully worded information about the reason for their recall and the process of the assessment (but not in such detail that they become distressed without the support of the screening staff being resent), and to have a breast care nurse or CNS available to answer any concerns they may have.
Brett, J.; 2005[9]	Anxiety	Not favourable	The results report that mammographic screening does not appear to create anxiety in women who are given a clear result after a mammogram and are subsequently placed on routine recall. However, women who have further investigations following their routine mammogram experience significant anxiety in the short term, and possibly in the long term.	None
Brewer, N. T.; 2007[10]	Anxiety	Not favourable	False-positive results were associated with generally more thoughts about breast cancer (including greater distress, anxiety, and worry and a greater perceived likelihood of receiving positive results for breast cancer in the future), but they had no consistent relationship to generalized anxiety or depression.	None
Broeders, M.; 2012[11]	Mortality	Favourable	After considering all published data from European studies, the reduction in breast cancer mortality associated with mammographic population-based service screening programmes is in the range of 25–31% for women invited for screening and 38–48% for women actually screened with sufficient follow-up time.	None
Elmore, J. G.; 2005 (40-49 y) [12]	Mortality; Overdiagnosis; False +ve; Pain/Discomfort	Not favourable	The benefit of screening women in their 40s is slower to appear and is somewhat less than that of women older than 50 years. Adjusted sensitivity increased with age from 69% in women aged 40 through 49 years to 83% in women aged 80 through 89 years.	None
Elmore, J. G.; 2005 (50-69y) [12]	Mortality; Overdiagnosis; False +ve; Pain/Discomfort	Favourable	Meta-analyses that included all trials demonstrated statistically significant reductions of 20% to 35% in mortality from breast cancer for women aged 50 to 69 years.	None
Elmore, J. G.; 2005 (70+ y) [12]	Mortality; Overdiagnosis; False +ve; Pain/Discomfort	Favourable	The benefit-to-harm ratio of screening increases as women age because screening accuracy improves and prevalence of breast cancer increases.	Encouraging individualized decisions may be especially appropriate for women older than 70 years.
Erpeldinger, S.; 2013[13]	Mortality	Not favourable	The all-cause death rate was not significantly reduced by screening when compared to the rate observed in unscreened women. However, mammography screening does not seem to induce excess mortality.	The modesty of the benefit size, which was estimated at 1 breast cancer death prevented in 10 years for every 2,000 women screened put into question the relevance of mass screening, and highlights the need for clear and complete information for the concerned patients.
Gabe, R.; 2005[14]	Mortality	Favourable	Finally, when the results of the longitudinal studies (i.e. the cohort and non-RCT comparative studies) were combined, a significant 26% reduction was observed with invitation and a significant 43% reduction was observed with screening.	None
Galit, W.; 2007[15]	Mortality; Overdiagnosis; Cost effectiveness/ QoL	Favourable	Regular mammography screening in olderwomen may be associated with an earlier-stage disease and lower breast cancer mortality.	Thus, we conclude that age should not be the only criteria for breast cancer screening attendance, and that decisions regarding national screening for breast cancer early detection in elderly women should be taken on the basis of individualized medical assessment as well as the woman's own preferences.
Gotzsche, P. C.; 2013[17]	Mortality	Not favourable	The effect of screening programmes, if any, is small and the balance between beneficial and harmful effects is very delicate.	We conclude that screening for breast cancer with mammography is unjustified.

Article and age group	Outcomes	Direction of conclusion	Conclusion statement	Recommendation Statement
Gotzsche, P. C.; 2006[18]	Mortality; Overdiagnosis; Surgery; Anxiety	Not favourable	The chance that a woman will benefit fromattending screening is small at best, and - if based on the randomised trials - ten times smaller than the risk that she may experience serious harm in terms of overdiagnosis.	Women, clinicians and policy makers should consider the trade-offs carefully when they decide whether or not to attend or support screening programmes.
Gotzsche, P. C.; 2009[19]	Mortality; Overdiagnosis; Surgery; Anxiety; Pain/Discomfort	Not favourable	Despite the shortcomings of the trials, screening appears to lower breast cancer mortality. However, the chance that a woman will benefit from attending screening is very small, and considerably smaller than the risk that she will experience harm. It is thus not clear whether screening does more good than harm.	Women, clinicians and policy makers should consider the trade-offs carefully when they decide whether or not to attend or support screening programs.
Gotzsche, P. C.; 2011 [20]	Mortality; Overdiagnosis;Surgery; Radiation Cancer; Anxiety	Not favourable	Despite the shortcomings of the trials, screening appears to lower breast cancer mortality. However, the chance that a woman will benefit from attending screening is very small, and considerably smaller than the risk that she may experience harm. It is thus not clear whether screening does more good than harm.	Women, clinicians and policy makers should consider the trade-offs carefully when they decide whether or not to attend or support screening programs.
Gotzsche, P. C.; 2011 [21]	Mortality; Overdiagnosis; Surgery; Anxiety; Pain/Discomfort	Not favourable	However, the chance that a woman will benefit from attending screening is very small, and considerably smaller than the risk that she may experience harm. It is thus not clearwhether screening does more good than harm.	Women, clinicians and policy makers should consider the trade-offs carefully when they decide whether or not to attend or support screening programs.
Gotzsche, P. C.;2000[16]	Mortality	Not favourable	Given that the size of the bias was similar to the estimated screening effect, screening appeared ineffective.	None
Green, B. B.; 2003[22]	Mortality; Overdiagnosis; False +ve	Favourable	The balance of the evidence still favors screening mammography in women aged 40 years and older at least every 2 years.	None
Hafslund, B.; 2009[23]	Anxiety; Cost effectiveness/ QoL	Not favourable	The literature search showed that psychological QoL aspects of mammography screening concerned many women. Some articles confirmed also disturbance in the physical and social domains of QoL.	None
Hamashima, C.; 2015 (40-49y) [24]	Mortality	Not favourable	In both screening methods, the NNI (number needed to invite) was higher in women aged 40–49 years than in women aged 50–70 years.	As there is still no standard established in Japan, the appropriateness of including women aged in their 40s in the NNI cannot be ascertained.
Hamashima, C.; 2015 (50+ y)[24]	Mortality	Favourable	In conclusion, the results of our analysis suggest that mammographic screening without clinical breast examination may afford higher benefits to women aged 50 years and over.	None
Harris, R.; 2011[25]	Mortality	Favourable	Current observational evidence shows that breast cancer screening in actual populations of women ages 50 to 69 reduces breast cancer mortality; the magnitude of the effect is probably smaller than predicted in the randomized controlled trials.	It is thus imperative that we continue monitoring population screening programs.
Hofvind, S.; 2012[26]	False +ve	Not favourable	The estimated cumulative risk of a false-positive screening result in women aged 50–69 undergoing 10 biennial screening tests varied from 8% to 21% in the three studies examined (pooled estimate 19.7%).	None
Jones, B. A.; 2003[27]	Mortality	Favourable	Notwithstanding some of the concerns about efficacy in younger women, these results suggest that young women benefit from repeat screening at least with respect to stage at diagnosis.	African American women should be screened annually beginning at age 40, with special efforts made to encourage regular screening in accordance with recommended intervals.
Jorgensen, K. J.; 2009[28]	Overdiagnosis	Not favourable	In populations offered organised screening for breast cancer, overdiagnosis (the detection of cancers that do not cause death or symptoms) was 52%.	None
Jorgensen, K. J.;2013[29]	Overdiagnosis	Not favourable	Our meta-analysis included data from five countries and we demonstrated that public mammography screening results in 52% overdiagnosis	A clear message must be sent that screening may not reduce the risk of dying from breast cancer, that attendance considerably increases the risk of receiving a breast cancer diagnosis and a mastectomy, and that abstaining from screening can therefore be a sensible choice for many women.

Article and age group	Outcomes	Direction of conclusion	Conclusion statement	Recommendation Statement
Lee, S. J.; 2013[30]	Mortality	Favourable	For breast cancer screening by mammography, it takes only 3.0 years to prevent one death per 5000 women screened, suggesting that for most women with a life expectancy less than three years, the harms likely outweigh the benefits. It takes 10.7 years to prevent one death from breast cancer for 1000 women screened, suggesting that for most women with a life expectancy greater than 10 years the benefits outweigh the harms.	Incorporating time lag estimates into screening guidelines would encourage a more explicit consideration of the risks and benefits of screening for breast and colorectal cancer.
Leung, G. M.; 2002[31]	Mortality	Not favourable	Population-based, mass mammographic screening is at best a misappropriation of resources and perhaps even harmful to patients.	Evidence is insufficient to justify population-based breast cancer screening by mammography for women in Hong Kong and other Asian populations with low breast cancer prevalence.
Mandelblatt, J.; 2003[32]	Cost effectiveness/ QoL	Favourable	Current estimates suggest that biennial breast cancer screening after age 65 years reduces mortality at reasonable costs for women without clinically significant comorbid conditions.	The consistent finding that screening after age 65 years reduces mortality from breast cancer at reasonable costs supports the general conclusion that screening should continue, especially if a woman is in good health.
Metsala, E.; 2012[33]	Anxiety	Not favourable	The studies found in this systematic review give implications for developing more effective interventions reducing BC worry in further examination of breast screening.	Until we completely know the aetiology of BC, early detection and screening as a part of it seems to be the best way.
Moss, S. M.; 2012[34]	Mortality	Favourable	The majority of studies suggested reductions in breast cancer mortality as a result of mammographic screening.	None
Njor, S.; 2012[35]	Mortality	Favourable	Using this approach we found a 31% reduction in breast cancer mortality among women offered screening at age 50–69 compared with women offered screening at age 50–59. a breast cancer mortality reduction of 26% after 6–11 years of follow-up seems to be the likely impact of the European service mammography screening programmes offered to women aged 50–69.	None
Olsen, O.; 2001[36]	Mortality	Not favourable	However, the review found that while several trials have been performed, most were of poor quality, and questions remain about the effectiveness of screening for lowering death rates due to breast cancer.	Women, clinicians and policy makers should consider these findings carefully when they decide whether or not to attend or support screening programs.
Pace, L. E.; 2014 (40-49 y)[37]	Mortality; Overdiagnosis; False +ve	Not favourable	Mammography screening appears to be associated with reduced breast cancer mortality ,but for some patients, the harms may outweigh the benefits.	To maximize the benefit ofmammography screening, decisions should be individualized based on patients' risk profiles and preferences.
Pace, L. E.; 2014 (50-59 y)[37]	Mortality; Overdiagnosis; False +ve	Not favourable	For every 10 000 women who get regular mammograms for the next 10 years, the number whose lives will be saved because of the mammogram is approximately 10 of 10 000 women aged 50 to 59 y. Among 10 000 women aged 50-year olds, 62 deaths would occur despite screening and 10 would be averted; 10, 000 women aged 50 years undergoing annual mammography for 10 years, approximately 6130 (95%CI,5800-6470) will have at least 1 false-positive result.	None
Pace, L. E.; 2014 (60-69 y)[37]	Mortality; Overdiagnosis; False +ve	Not favourable	For every 10 000 women who get regular mammograms for the next 10 years, the number whose lives will be saved because of the mammogram is approximately 42 of 10 000 women aged 60 to 69y. Among 10 000 women aged 60-year-olds, 88 deaths would occur despite screening and 42 would be averted. 10, 000 women aged 60 years undergoing annual mammography for 10 years,approximately 4970 (95% CI, 4780-5150) will have at least 1 false-positive result.	None
Paesmans, M.; 2010[38]	Mortality; Overdiagnosis; False +ve ; Surgery	Not favourable	The probability of not dying from breast cancer within 15 years of screening for a 65-year-old woman is estimated to be in the range 98.85–99.11%, compared with 98.73% for a woman who does not undergo screening. These benefits should be balanced against the possible disadvantages of screening, including false positive results, overdiagnosis and overtreatment.	However, every woman who is offered screening should be as accurately informed as possible about the potential disadvantages as well as the potential benefits of screening, in order to allow her to make a truly informed decision.

Article and age group	Outcomes	Direction of conclusion	Conclusion statement	Recommendation Statement
Puliti, D.; 2012[39]	Overdiagnosis	Not favourable	The most plausible estimates of overdiagnosis range from 1% to 10%.	None
Ravert, P. K.; 2010[40]	False +ve	Favourable	This analysis supports yearly screening with mammography for women in the general population beginning at the age of 40.	Women in the general population who do not fall into the categories of high risk or dense breast tissue should receive a yearly mammogram starting at age 40.
Ringash, J.;2001[41]	Mortality	Not favourable	Screening mammography offers the potential for significant benefits in addition to mortality reduction, including early diagnosis, less aggressive therapy and improved cosmetic results. However, the risks of screening include increased biopsy rates and the psychological effects of false reassurance or false-positive results.	Current evidence regarding the effectiveness of screening mammography does not suggest the inclusion of the manoeuvre in, or its exclusion from, the periodic health examination of women aged 40–49 at average risk of breast cancer (grade C recommendation). Upon reaching the age of 40, Canadian women should be informed of the potential benefits and risks of screening mammography and assisted in deciding at what age they wish to initiate the manoeuvre.
Royak-Schaler, R.; 2002[42]	Mortality	Favourable	In 1995, both Feig and Smith [117] defended the routine mammography screening of women in the 40–49-year age range, and both of these investigators also stressed the need for shorter intervals between screenings for women in this age group [117,118]. However, despite the advocacy by some investigators the debate over the merits of mammographic screening in women younger than 50 years continues, and seems unlikely to be resolved to everyone's satisfaction any time soon.	Even though rates of mammography screening have increased significantly for AA women since the 1980s, it may be that the nationally recommended guidelines are suboptimal for their needs given the early onset and aggressive nature of their breast cancers, as suggested previously by Rose and Royak-Schaler [46] and Jones et al. [41] in their review presented at the year 2000 Summit Evaluating Research on Breast Cancer in AA women.
Scheel, J. R.; 2015[43]	Overdiagnosis; False +ve	Favourable	Although not perfect, mammography is still the only screening test whose ability to reduce breast cancer mortality has been proven. The evidence supports long-term benefits for women of any breast density with regular mammographic screening, and women with dense breasts should be urged to seek digital mammography for screening.	Screening ultrasound is recommended only for women at greater than a 20% lifetime risk for developing breast cancer who cannot undergo an MRI examination. Our study results support these recommendations.
Schopper, D.; 2009[44]	Mortality	Favourable	The evidence emerging from long-standing breast cancer screening programmes shows a clear, albeit variable impact on breast cancer mortality.	None
Suzuki, A.; 2014[45]	Mortality; Overdiagnosis; False +ve; Surgery; Radiation Cancer	Favourable	The effect of the mortality reduction in women aged 40–49 y was almost the same level as that of women aged 50–59 years;	None
Tange, U. B.; 2002[46]	False +ve; Surgery	Favourable	A significant reduction in tumour stage and an increase in the incidence of DCIS are observed both in the population offered screening, and more pronounced in the cases actually diagnosed by mammography screening compared to clinically diagnosed cases. Where reported, the impact of mammography screening on treatment is a reduction of the proportion of breast cancer patients treated with mastectomy, and a reduction of the proportion needing adjuvant systemic therapy.	None
Walter, L. C. ; 2014 (Biennial)[47]	Mortality; Overdiagnosis; False +ve; Cost effectiveness/ QoL	Favourable	In general, retrospective cohort and case-control studies found reduced breast cancer mortality associated with mammographic detection of breast cancer in women aged 75 years and older. Studies also show that it is cost effective to conduct biennial screening mammography up to a life expectancy of 9.5 years. Among women aged 75y and older who undergo biennial screening, the cumulative probability of a false-positive mammogram over 10 years ranges from 14% to 27%. This risk nearly doubles if women are screened annually.	When counseling these older women, it should be explained that it is not known if mammography decreases the risk of dying from breast cancer in women aged 75 years and older and a choice should be made whether to continue screening.

Article and age group	Outcomes	Direction of conclusion	Conclusion statement	Recommendation Statement
Walter, L. C. ; 2014 (Annual) [47]	False +ve	Not favourable	Among women aged 75y and older who undergo biennial screening, the cumulative probability of a false-positive mammogram over 10 years ranges from 14% to 27%. This risk nearly doubles if women are screened annually.	None
Yarbrough, S. S.;2004[48]	Mortality	Favourable	Although no evidence exists that screening may affect overall life expectancy, evidence confirms that health status is heterogeneous in this population and that many older women could tolerate screening and treatment	Until then, each woman's right to participate in screening or treatment should be ensured through shared, informed, autonomous decision making.
Yoo, K. B.; 2013[49]	Cost effectiveness/ QoL	Not favourable	The results show that mammography mass screening is not cost-effective in Asian countries, unlike Western countries, due to breast cancer incidence rate and racial characteristics issues.	The countries that have a low breast cancer incidence rate, such as Asian countries, should act prudently when implementing mammography as the reference test targeting the general population. Other screening methods such as clinical breast examination could be a possible alternative.
Zelle, S. G.; 2013[50]	Cost effectiveness/ QoL	Favourable	The limited evidence base suggests that screening strategies may be economically attractive in LMICs – yet there is very little evidence to provide specific recommendations (on screening by mammography vs. CBE, the frequency of screening, or the target population).	None
