# Supplementary material for: Conclusions in systematic reviews of mammography for breast cancer screening and associations with review design and author characteristics
Source: Syst Rev. 2017 May 22;6:105. doi: 10.1186/s13643-017-0495-6 (PMC5441061; doi:10.1186/s13643-017-0495-6)
Supplement: Supplementary file 4 — Included systematic reviews. References for all included systematic reviews. (PDF 229 kb) [file 13643_2017_495_MOESM4_ESM.pdf]

Included Systematic Reviews

1. Armstrong K, Moye E, Williams S, et al. Screening mammography in women 40 to 49 years of age: a systematic review for the American College of Physicians. Annals of Internal Medicine 2007;**146**(7):516-26.
2. Autier P, Boniol M, Middleton R, et al. Advanced breast cancer incidence following population-based mammographic screening. Annals of Oncology 2011;**22**(8):1726-35.
3. Baker S, Wall M, Bloomfield A. Breast cancer screening for women aged 40 to 49 years--what does the evidence mean for New Zealand? The New Zealand Medical Journal 2005;**118**(1221):U1628.
4. Baker S, Wall M, Bloomfield A. What is the most appropriate breast-cancer screening interval for women aged 45 to 49 years in New Zealand? The New Zealand Medical Journal 2005;**118**(1221):U1636.
5. Barratt AL, Les Irwig M, Glasziou PP, et al. Benefits, harms and costs of screening mammography in women 70 years and over: a systematic review. The Medical Journal of Australia 2002;**176**(6):266-71.
6. Biesheuvel C, Barratt A, Howard K, et al. Effects of study methods and biases on estimates of invasive breast cancer overdetection with mammography screening: a systematic review. The Lancet Oncology 2007;**8**(12):1129-38.
7. Bond M, Pavey T, Welch K, et al. Psychological consequences of false-positive screening mammograms in the UK. Evidence-based Medicine 2013;**18**(2):54-61.
8. Bond M, Pavey T, Welch K, et al. Systematic review of the psychological consequences of false-positive screening mammograms. Health Technology Assessment 2013;**17**(13):1-170, v-vi.
9. Brett J, Bankhead C, Henderson B, et al. The psychological impact of mammographic screening. A systematic review. Psycho-oncology 2005;**14**(11):917-38.
10. Brewer NT, Salz T, Lillie SE. Systematic review: the long-term effects of false-positive mammograms. Annals of Internal Medicine 2007;**146**(7):502-10.
11. Broeders M, Moss S, Nystrom L, et al. The impact of mammographic screening on breast cancer mortality in Europe: a review of observational studies. Journal of Medical Screening 2012;**19 Suppl 1**:14-25.
12. Elmore JG, Armstrong K, Lehman CD, et al. Screening for breast cancer. JAMA 2005;**293**(10):1245-56.
13. Erpeldinger S, Fayolle L, Boussageon R, et al. Is there excess mortality in women screened with mammography: a meta-analysis of non-breast cancer mortality. Trials 2013;**14**:368.
14. Gabe R, Duffy SW. Evaluation of service screening mammography in practice: the impact on breast cancer mortality. Annals of Oncology 2005;**16 Suppl 2**:ii153-62.
15. Galit W, Green MS, Lital KB. Routine screening mammography in women older than 74 years: a review of the available data. Maturitas 2007;**57**(2):109-19.

16. Gotzsche PC, Olsen O. Is screening for breast cancer with mammography justifiable? Lancet 2000;**355**(9198):129-34.

17. Gotzsche PC, Jorgensen KJ. Screening for breast cancer with mammography. The Cochrane Database of Systematic Reviews 2013(6):cd001877.

18. Gotzsche PC, Nielsen M. Screening for breast cancer with mammography. The Cochrane Database of Systematic Reviews 2006(4):cd001877.

19. Gotzsche PC, Nielsen M. Screening for breast cancer with mammography. The Cochrane Database of Systematic Reviews 2009(4):cd001877.

20. Gotzsche PC, Nielsen M. Screening for breast cancer with mammography. The Cochrane Database of Systematic Reviews 2011(1):cd001877.

21. Gotzsche PC. Relation between breast cancer mortality and screening effectiveness: systematic review of the mammography trials. Danish Medical Bulletin 2011;**58**(3):A4246.

22. Green BB, Taplin SH. Breast cancer screening controversies. The Journal of the American Board of Family Practice 2003;**16**(3):233-41.

23. Hafslund B, Nortvedt MW. Mammography screening from the perspective of quality of life: a review of the literature. Scandinavian Journal of Caring Sciences 2009;**23**(3):539-48.

24. Hamashima C, Ohta K, Kasahara Y, et al. A meta-analysis of mammographic screening with and without clinical breast examination. Cancer Science 2015;**106**(7):812-8.

25. Harris R, Yeatts J, Kinsinger L. Breast cancer screening for women ages 50 to 69 years a systematic review of observational evidence. Preventive Medicine 2011;**53**(3):108-14.

26. Hofvind S, Ponti A, Patnick J, et al. False-positive results in mammographic screening for breast cancer in Europe: a literature review and survey of service screening programmes. Journal of Medical Screening 2012;**19 Suppl 1**:57-66.

27. Jones BA, Patterson EA, Calvocoressi L. Mammography screening in African American women: evaluating the research. Cancer 2003;**97**(1 Suppl):258-72.

28. Jorgensen KJ, Gotzsche PC. Overdiagnosis in publicly organised mammography screening programmes: systematic review of incidence trends. BMJ 2009;**339**:b2587.

29. Jorgensen KJ. Mammography screening. Benefits, harms, and informed choice. Danish Medical Journal 2013;**60**(4):B4614.

30. Lee SJ, Boscardin WJ, Stijacic-Cenzer I, et al. Time lag to benefit after screening for breast and colorectal cancer: meta-analysis of survival data from the United States, Sweden, United Kingdom, and Denmark. BMJ 2013;**346**:e8441.

31. Leung GM, Lam TH, Thach TQ, et al. Will screening mammography in the East do more harm than good? American Journal of Public Health 2002;**92**(11):1841-6.

32. Mandelblatt J, Saha S, Teutsch S, et al. The cost-effectiveness of screening mammography beyond age 65 years: a systematic review for the U.S. Preventive Services Task Force. Annals of Internal Medicine 2003;**139**(10):835-42.

33. Metsala E, Pajukari A, Aro AR. Breast cancer worry in further examination of mammography screening--a systematic review. Scandinavian Journal of Caring Sciences 2012;**26**(4):773-86.

34. Moss SM, Nystrom L, Jonsson H, et al. The impact of mammographic screening on breast cancer mortality in Europe: a review of trend studies. Journal of Medical Screening 2012;**19 Suppl 1**:26-32.

35. Njor S, Nystrom L, Moss S, et al. Breast cancer mortality in mammographic screening in Europe: a review of incidence-based mortality studies. Journal of Medical Screening 2012;**19 Suppl 1**:33-41.

36. Olsen O, Gotzsche PC. Screening for breast cancer with mammography. The Cochrane Database of Systematic Reviews 2001(4):cd001877.

37. Pace LE, Keating NL. A systematic assessment of benefits and risks to guide breast cancer screening decisions. JAMA 2014;**311**(13):1327-35.

38. Paesmans M, Ameye L, Moreau M, et al. Breast cancer screening in the older woman: an effective way to reduce mortality? Maturitas 2010;**66**(3):263-7.

39. Puliti D, Duffy SW, Miccinesi G, et al. Overdiagnosis in mammographic screening for breast cancer in Europe: a literature review. Journal of Medical Screening 2012;**19 Suppl 1**:42-56.

40. Ravert PK, Huffaker C. Breast cancer screening in women: An integrative literature review. Journal of the American Academy of Nurse Practitioners 2010;**22**(12):668-73.

41. Ringash J. Preventive health care, 2001 update: screening mammography among women aged 40-49 years at average risk of breast cancer. CMAJ 2001;**164**(4):469-76.

42. Royak-Schaler R, Rose DP. Mammography screening and breast cancer biology in African American women--a review. Cancer Detection and Prevention 2002;**26**(3):180-91.

43. Scheel JR, Lee JM, Sprague BL, et al. Screening ultrasound as an adjunct to mammography in women with mammographically dense breasts. American Journal of Obstetrics and Gynecology 2015;**212**(1):9-17.

44. Schopper D, de Wolf C. How effective are breast cancer screening programmes by mammography? Review of the current evidence. European Journal of Cancer 2009;**45**(11):1916-23.

45. Suzuki A, Ishida T, Ohuchi N. Controversies in breast cancer screening for women aged 40-49 years. Japanese Journal of Clinical Oncology 2014;**44**(7):613-8.

46. Tange UB, Jensen MB, Vejborg IM, et al. Clinical impact of introduction of mammography screening in a non-screening country with special reference to the Copenhagen service mammography screening programme. Scandinavian Journal of Surgery 2002;**91**(3):293-303.

47. Walter LC, Schonberg MA. Screening mammography in older women: a review. JAMA 2014;**311**(13):1336-47.

48. Yarbrough SS. Older women and breast cancer screening: research synthesis. Oncology Nursing Forum 2004;**31**(1):E9-15.

49. Yoo KB, Kwon JA, Cho E, et al. Is mammography for breast cancer screening cost-effective in both Western and asian countries?: results of a systematic review. Asian Pacific Journal of Cancer Prevention 2013;**14**(7):4141-9.

50. Zelle SG, Baltussen RM. Economic analyses of breast cancer control in low- and middle-income countries: a systematic review. Systematic Reviews 2013;**2**:20.
