# Supplementary material for: Conclusions in systematic reviews of mammography for breast cancer screening and associations with review design and author characteristics
Source: Syst Rev. 2017 May 22;6:105. doi: 10.1186/s13643-017-0495-6 (PMC5441061; doi:10.1186/s13643-017-0495-6)
Supplement: Supplementary file 5 — Associations between systematic review characteristics and conclusions in 5 conclusions of studies that included women aged 70 years and older. (PDF 193 kb) [file 13643_2017_495_MOESM5_ESM.pdf]

Additional Table 3. Associations between systematic review characteristics and conclusions in 5 conclusions of studies that included women aged 70 years and older.

Characteristics	Number of conclusions	Proportion of favourable conclusions (%)	p-value (chi-square test)
**Corresponding author**			
Non-clinical	2	2 (100%)	$p=0.36; X^2=0.83$
Clinical	3	2 (67%)	
**Competing interests**			
Declared none	3	2 (67%)	$p=0.66; X^2=0.83$
No statement	1	1 (100%)	
Declared	1	1 (100%)	
**Type of evidence**			
RCT only	0	NA	NA
RCT and non-RCT	1	1 (100%)	
Non-RCT only	4	3 (75%)	
Cost-effectiveness	0	NA	
**Outcome measures**			
Did not include harms	1	1 (100%)	$p=0.58; X^2=0.31$
Included harms or overdiagnosis	4	3 (75%)	
**Meta-analysis**			
Yes	0	NA	NA
No	5	4 (80%)	
