# Supplementary material for: Conclusions in systematic reviews of mammography for breast cancer screening and associations with review design and author characteristics
Source: Syst Rev. 2017 May 22;6:105. doi: 10.1186/s13643-017-0495-6 (PMC5441061; doi:10.1186/s13643-017-0495-6)
Supplement: Supplementary file 6 — Included systematic review conclusions in detail. (PDF 774 kb) [file 13643_2017_495_MOESM6_ESM.pdf]

**Additional Table 4.** Included systematic review conclusion characteristics in detail.

| | Type of Evidence | | | | Outcome Measures | | | | | | | | Authors | | | Competing Interests | | | Age Groups | | | | Type of Analysis | | Conclusion | |
|---|---|---|---|---|---|---|---|---|---|---|---|---|---|---|---|---|---|---|---|---|---|---|---|---|---|---|
| **Conclusion by systematic review and age group** | **Controlled trials** | **Both** | **Non-controlled studies** | **Cost effectiveness** | **Mortality** | **Over-diagnosis** | **False positive** | **Unnecessary treatment** | **Radiation cancer** | **Anxiety/ Worry** | **Pain/ discomfort** | **Cost Effectiveness** | **Clinician corresponding** | **Mixed author group** | **Non-clinician corresp.** | **No statement** | **Declared none** | **Declared** | **Not specified/ All ages** | **Up to 49** | **50-69** | **70 and over** | **Meta-analysis** | **No meta-analysis** | **Favourable** | **Not favourable** |
| Armstrong, K.; 2007[1] (40-49y) | | X | | | X | X | X | | X | | X | | X | X | | | | X | | X | | | | X | | X |
| Autier, P.; 2011[2] | | | X | | X | | | | | | | | | | X | | X | | X | | | | | X | | X |
| Baker, S.; 2005 [3] | X | | | | X | X | X | | X | | | X | | X | X | X | | | | X | | | | X | | X |
| Biesheuvel,C.; 2007[6] (40-49y) | | X | | | | X | | | | | | | | | X | | X | | | X | | | | X | | X |
| Biesheuvel,C.; 2007[6] (50-59y) | | X | | | | X | | | | | | | | | X | | X | | | | X | | | X | | X |
| Biesheuvel,C.; 2007[6] (60-69y) | | X | | | | X | | | | | | | | | X | | X | | | | X | | | X | | X |
| Bond, M.; 2013[7] | | X | | | | | | | | X | | | | | X | | X | | X | | | | | X | | X |
| Bond, M.; 2013[8] | | X | | | | | | | | X | | | | | X | | X | | X | | | | X | | | X |
| Brett, J.; 2005[9] | | | X | | | | | | | X | | | | | X | | X | | | | X | | | X | | X |
| Brewer, N. T.; 2007[10] | | | X | | | | | | | X | | | | | X | | X | | X | | | | X | | | X |
| Elmore, J. G.; 2005[12] (40-49y) | | X | | | X | X | X | | | | X | | X | X | | | | X | | X | | | | X | | X |
| Erpeldinger, S.; 2013[13] | X | | | | X | | | | | | | | | X | X | | X | | X | | | | X | | | X |
| Gotzsche, P. C.;2000[16] | X | | | | X | | | | | | | | | | X | X | | | X | | | | X | | | X |
| Gotzsche, P. C.; 2013[17] | | X | | | X | X | | X | | X | | | | | X | | X | | X | | | | | X | | X |
| Gotzsche, P. C.; 2006[18] | | X | | | X | X | | X | | X | X | | | | X | | X | | | | X | | X | | | X |
| Gotzsche, P. C.; 2009[19] | | X | | | X | X | | X | X | X | | | | | X | | X | | X | | | | | X | | X |
| Gotzsche, P. C.; 2011[20] | | X | | | X | X | | X | | X | X | | | | X | | X | | | | X | | X | | | X |
| Gotzsche, P. C.; 2011[21] | | X | | | X | | | | | | | | | | X | | X | | X | | | | | X | | X |
| Hafslund, B.; 2009[23] | | | X | | | | | | | X | | X | X | X | | X | | | X | | | | | X | | X |
| Hamashima, C.; 2015[24] (50+y) | X | | | | X | | | | | | | | X | X | | | X | | | | X | | X | | | X |
| Hofvind, S.; 2012[26] | | | X | | | | X | | | | | | X | X | | X | | | | | X | | | X | | X |
| Jorgensen, K. J.; 2009[28] | | | X | | | X | | | | | | | | | X | | X | | X | | | | X | | | X |
| Jorgensen, K. J.;2013[29] | | X | | | | X | | | | | | | | | X | X | | | X | | | | | X | | X |
| Leung, G. M.; 2002[31] | X | | | | X | | | | | | | | | | X | X | | | | | X | | X | | | X |
| Metsala, E.; 2012[33] | | | X | | | | | | | X | | | | X | X | X | | | X | | | | X | | | X |
| Olsen, O.; 2001[36] | X | | | | X | | | | | | | | | | X | | X | | | | X | | X | | | X |
| Pace, L. E.; 2014[37] (40-49y) | | X | | | X | X | X | | | | | | | X | X | | X | | | X | | | | X | | X |
| Pace, L. E.; 2014[37] (50-59y) | | X | | | X | X | X | | | | | | | X | X | | X | | | | X | | | X | | X |
| Pace, L. E.; 2014[37] (60-69y) | | X | | | X | X | X | | | | | | | X | X | | X | | | | X | | | X | | X |
| Paesmans, M.; 2010[38] | X | | | | X | X | X | X | | | | | | | X | | X | | | | X | | | X | | X |
| Puliti, D.; 2012[39] | | | X | | | X | | | | | | | | X | X | X | | | X | | | | | X | | X |
| Ringash, J.;2001[41] | | X | | | X | | | | | | | | X | X | | X | | | | X | | | X | | | X |
| Walter, L. C. ; 2014[47] (Biennial) | | | X | | | | X | | | | | | X | | | | X | | | | | X | | X | | X |
| Yoo, K. B.; 2013[49] | | | | X | | | | | | | | X | | | X | X | | | X | | | | | X | | X |
| Armstrong, K.; 2007[1] (50y+) | | X | | | X | X | X | | X | | X | | X | X | | | | X | | | X | | | X | X | |
| Baker, S.; 2005[4] | X | | | | X | | | | | | | | | X | X | X | | | | X | | | | X | X | |
| Barratt, A. L.; 2002[5] | | | X | | | | | | | | | X | | X | X | | X | | | | | X | | X | X | |
| Broeders, M.; 2012[11] | | | X | | X | | | | | | | | | | X | X | | | | | X | | X | | X | |
| Elmore, J. G.; 2005[12] (60-69y) | | X | | | X | X | X | | | | X | | X | X | | | | X | | | X | | | X | X | |
| Elmore, J. G.; 2005[12] (70+) | | X | | | X | X | X | | | | X | | X | X | | | | X | | | | X | | X | X | |
| Gabe, R.; 2005[14] | | | X | | X | | | | | | | | | | X | | X | | X | | | | X | | X | |
| Galit, W.; 2007[15] | | | X | | X | X | | | | | | X | | | X | X | | | | | | X | | X | X | |
| Green, B. B.; 2003[22] | X | | | | X | X | X | | | | | | | | X | X | | | X | | | | | X | X | |
| Hamashima, C.; 2015[24] (40-49y) | X | | | | X | | | | | | | | X | X | | | X | | | X | | | X | | X | |
| Harris, R.; 2011[25] | | | X | | X | | | | | | | | | | X | X | | | | | X | | | X | X | |
| Jones, B. A.; 2003[27] | | X | | | X | | | | | | | | | | X | | | X | X | | | | | X | X | |
| Lee, S. J.; 2013[30] | X | | | | X | | | | | | | | X | X | | | | X | | | X | | X | | X | |
| Mandelblatt, J.; 2003[32] | | | | X | | | | | | | | X | X | X | | | X | | | | X | | | X | X | |
| Moss, S. M.; 2012[34] | | | X | | X | | | | | | | | | X | X | X | | | X | | | | | X | X | |
| Njor, S.; 2012[35] | | | X | | X | | | | | | | | | X | X | X | | | | | X | | | X | X | |
| Ravert, P. K.; 2010[40] | | | X | | | | X | | | | | | X | | | | X | | X | | | | | X | X | |
| Royak-Schaler, R.; 2002[42] | | X | | | X | | | | | | | | | | X | X | | | | X | | | | X | X | |
| Scheel, J. R.; 2015[43] | | | X | | | X | X | | | | | | X | X | | | | X | | | X | | | X | X | |
| Schopper, D.; 2009[44] | | | X | | X | | | | | | | | | X | X | | X | | X | | | | | X | X | |
| Suzuki, A.; 2014[45] | X | | | | X | X | X | X | X | | | | X | | | | X | | | X | | | | X | X | |
| Tange, U. B.; 2002[46] | | X | | | | | X | X | | | | | X | X | | X | | | | | X | | | X | X | |
| Walter, L. C. ; 2014[47] (Annual) | | | X | | X | X | X | | | | | X | X | | | | X | | | | | X | | X | X | |
| Yarbrough, S. S.;2004[48] | | X | | | X | | | | | | | | X | | | | X | | | | X | | | X | X | |
| Zelle, S. G.; 2013[50] | | | | X | | | | | | | | X | | | X | | X | | X | | | | | X | X | |
