# Supplementary material for: Conclusions in systematic reviews of mammography for breast cancer screening and associations with review design and author characteristics
Source: Syst Rev. 2017 May 22;6:105. doi: 10.1186/s13643-017-0495-6 (PMC5441061; doi:10.1186/s13643-017-0495-6)
Supplement: Supplementary file 7 — Associations between systematic review characteristics and conclusions in 22 conclusions of studies that did not specify age group or specified all ages. (PDF 193 kb) [file 13643_2017_495_MOESM7_ESM.pdf]

Additional Table 1. Associations between systematic review characteristics and conclusions in 22 conclusions of studies that did not specify age group or specified all ages.

Characteristics	Number of conclusions	Proportion of favourable conclusions (%)	p-value (chi-square test)
**Corresponding author**			
Non-clinical	20	6 (30%)	$p=0.56; X^2=0.34$
Clinical	2	1 (50%)	
**Competing interests**			
Declared none	13	4 (31%)	$p=0.31; X^2=2.32$
No statement	8	2 (25%)	
Declared	1	1 (100%)	
**Type of evidence**			
RCT only	3	1 (33%)	$p=0.66; X^2=1.61$
RCT and non-RCT	7	1(14%)	
Non-RCT only	10	4 (40%)	
Cost-effectiveness	2	1 (50%)	
**Outcome measures**			
Did not include harms	10	5 (50%)	$p=0.10; X^2=2.79$
Included harms or overdiagnosis	12	2 (16%)	
**Meta-analysis**			
Yes	7	1 (14%)	$p=0.23; X^2=1.46$
No	15	6 (40%)	
