# Supplementary material for: Conclusions in systematic reviews of mammography for breast cancer screening and associations with review design and author characteristics
Source: Syst Rev. 2017 May 22;6:105. doi: 10.1186/s13643-017-0495-6 (PMC5441061; doi:10.1186/s13643-017-0495-6)
Supplement: Supplementary file 8 — Associations between systematic review characteristics and conclusions in 10 conclusions of studies that included women aged up to 49 years. (PDF 193 kb) [file 13643_2017_495_MOESM8_ESM.pdf]

Additional Table 2. Associations between systematic review characteristics and conclusions in 10 conclusions of studies that included women aged up to 49 years.

Characteristics	Number of conclusions	Proportion of favourable conclusions (%)	p-value (chi-square test)
**Corresponding author**			
Non-clinical	5	2 (40%)	$p=1.00; X^2=0.00$
Clinical	5	2 (40%)	
**Competing interests**			
Declared none	4	2 (50%)	$p=0.44; X^2=1.67$
No statement	4	2 (50%)	
Declared	2	0 (0%)	
**Type of evidence**			
RCT only	4	3 (75%)	NA
RCT and non-RCT	6	1 (16%)	
Non-RCT only	0	NA	
Cost-effectiveness	0	NA	
**Outcome measures**			
Did not include harms	4	3 (75%)	$p=0.065; X^2=3.40$
Included harms or overdiagnosis	6	1 (16%)	
**Meta-analysis**			
Yes	2	1 (50%)	$p=0.75; X^2=0.10$
No	8	3 (38%)	
